# Membrane Elastic Properties during Neural Precursor Cell Differentiation

**DOI:** 10.3390/cells9061323

**Published:** 2020-05-26

**Authors:** Juliana Soares, Glauber R. de S. Araujo, Cintia Santana, Diana Matias, Vivaldo Moura-Neto, Marcos Farina, Susana Frases, Nathan B. Viana, Luciana Romão, H. Moysés Nussenzveig, Bruno Pontes

**Affiliations:** 1 Instituto de Ciências Biomédicas, Universidade Federal do Rio de Janeiro, Rio de Janeiro, RJ 21941-902, Brazil; juli.soares.m@gmail.com (J.S.); cfsfranca10@gmail.com (C.S.); dimtias@gmail.com (D.M.); vivaldomouraneto@gmail.com (V.M.-N.); marcos.farina.souza@gmail.com (M.F.); luromao@gmail.com (L.R.); 2 Centro Nacional de Biologia Estrutural e Bioimagem (CENABIO), Universidade Federal do Rio de Janeiro, Rio de Janeiro, RJ 21941-902, Brazil; nathanbessaviana@gmail.com; 3 Instituto de Biofísica Carlos Chagas Filho, Universidade Federal do Rio de Janeiro, Rio de Janeiro, RJ 21941-902, Brazil; glauber@biof.ufrj.br (G.R.d.S.A.); susanafrases@biof.ufrj.br (S.F.); 4 Instituto Estadual do Cérebro Paulo Niemeyer, Secretaria de Estado de Saúde, Rio de Janeiro, RJ 20231-092, Brazil; 5 Instituto de Física, Universidade Federal do Rio de Janeiro, Rio de Janeiro, RJ 21941-942, Brazil

**Keywords:** optical tweezers, membrane-cytoskeleton complex, membrane elastic properties, membrane tension, neural precursor cells, neurons, astrocytes, oligodendrocytes

## Abstract

Neural precursor cells differentiate into several cell types that display distinct functions. However, little is known about how cell surface mechanics vary during the differentiation process. Here, by precisely measuring membrane tension and bending modulus, we map their variations and correlate them with changes in neural precursor cell morphology along their distinct differentiation fates. Both cells maintained in culture as neural precursors as well as those plated in neurobasal medium reveal a decrease in membrane tension over the first hours of culture followed by stabilization, with no change in bending modulus. During astrocyte differentiation, membrane tension initially decreases and then increases after 72 h, accompanied by consolidation of glial fibrillary acidic protein expression and striking actin reorganization, while bending modulus increases following observed alterations. For oligodendrocytes, the changes in membrane tension are less abrupt over the first hours, but their values subsequently decrease, correlating with a shift from oligodendrocyte marker O4 to myelin basic protein expressions and a remarkable actin reorganization, while bending modulus remains constant. Oligodendrocytes at later differentiation stages show membrane vesicles with similar membrane tension but higher bending modulus as compared to the cell surface. Altogether, our results display an entire spectrum of how membrane elastic properties are varying, thus contributing to a better understanding of neural differentiation from a mechanobiological perspective.

## 1. Introduction

The surface of all mammalian cells is composed of the plasma membrane cushioned underneath by a cortical actomyosin cytoskeleton. This pair of structures forms the membrane–cytoskeleton complex, a key regulator of several cellular processes, ranging from shape control and cell migration to molecule presentation and signaling [1,2]. The plasma membrane is the direct interface between the cytoplasm and the extracellular matrix [3,4], while the cortical cytoskeleton, also known as cell cortex, is a dynamic actomyosin meshwork that gives support to the plasma membrane [1,2]. The membrane-cytoskeleton complex exerts and reacts against forces owing to its elastic properties [1,2,4]. For brevity, we shall just refer to this complex as “cell membrane” (CM).

Over the years, different micromanipulation tools have been employed to exert forces on CMs so as to characterize their elastic responses [5]. Membrane tether pulling assays using optical tweezers (OT) [6,7] or atomic force microscopy (AFM) [8,9] have been used to extract nanotubes or tethers from CMs in order to determine these properties. By measuring both the equilibrium force and tether radius, the cell membrane surface tension (CMT - $\sigma_{eff}$) and cell membrane bending modulus (CMBM - $\kappa_{eff}$) have been determined for different cell types [7,10,11,12,13,14,15]. CMT and CMBM result from joint contributions of cytoskeleton architecture, membrane composition and membrane-cytoskeleton attachment [14]. Moreover, these elastic properties (particularly CMT), as well as their changes, have been characterized as important regulators of cellular behaviors, especially regarding shape changes and force production [14,16]. Furthermore, it has been shown [13] that membrane elastic properties are correlated to cell function.

During development, neural precursor cells (NPCs), which collectively describe the mixed population of neural stem cells and neural progenitor cells, give rise to all neurons, astrocytes and oligodendrocytes of the mammalian central nervous system (CNS) [17]. These three cell types are extremely different, not only in their morphological features but also in their functions. Neurons are highly anisotropic cells, with relatively quiescent and compact cell bodies (soma) containing the cell nucleus and dynamic protrusions (axons and dendrites), both susceptible to large structural changes [18]. Astrocytes are remarkably dynamic, constantly modifying their morphology during migration or when interacting with neurons [19]. Oligodendrocytes extend many protrusions that can ultimately form myelin sheaths, wrapping around axons to promote the fast saltatory conduction of action potentials [20]. Several studies have revealed the molecular mechanisms that govern the path of NPC differentiation and fate specification. Diverse growth factors and cytokines, together with epigenetic alterations including DNA methylation, histone modifications, and non-coding RNAs have already been described as key elements in this regard [21], as well as gene expression changes [22]. However, little is known about how the CM elastic properties of NPCs vary over the course of their differentiation nor the correlation between such variations and the final morphological phenotype of differentiated cells.

In the present work, we combined OT-based tether extraction experiments with measurements of tether radius using scanning electron microscopy (SEM), as well as fluorescence microscopy observations, to investigate the roles of the cytoskeleton on the CM elastic properties of NPCs along their distinct differentiation fates. Our aim was to map and collect experimental evidence that may provide a basis for future studies on how modifications in CM elastic properties correlate with shape and phenotype changes, ultimately influencing NPC differentiation.

## 2. Materials and Methods

### 2.1. Animals

For NPC cultures, embryonic (E14; 14-day embryonic) Swiss mice, obtained from pregnant females, were used. Mice were maintained at the Institute of Biomedical Sciences, Federal University of Rio de Janeiro. Animal husbandry and experimental procedures were performed in accordance with and approved by the Ethics Committee of the Health Sciences Center, Federal University of Rio de Janeiro (Protocol Number: 001-16). 

### 2.2. Cell Cultures

NPC cultures were obtained according to a modified protocol [23]. Briefly, cerebral hemispheres from 14-day-old mouse embryos were isolated and maintained in phosphate-buffered saline (PBS), meninges were removed and the ganglionic eminences and developing cerebral cortices were dissected out, minced and mechanically dissociated. Aggregates were separated by decantation and cells were obtained by centrifugation at 180× *g* for 5 min. The pellet was resuspended in Dulbecco’s Modified Eagle’s (DMEM) F-12 medium containing 0.6% glucose, N2, G5 (with FGF and EGF) and B27 supplements, 2 mM L-glutamine, 5 mM HEPES, 0,11% NaHCO_3_, and 1% penicillin/streptomycin (all from Invitrogen, Thermo Fisher, Carlsbad, CA, USA). The NPCs were cultured as neurospheres for 5 days. Then, neurospheres or dissociated NPCs were resuspended in the same medium that maintains their stemness, as described above, and plated onto coverslips or glass bottom dishes previously coated with 0.01% poly-L-lysine (Sigma-Aldrich, St. Louis, MO, USA) for 2 h. Dissociated NPCs were also placed in Neurobasal media supplemented with 2 mM L-glutamine, 1% penicillin/streptomycin and B27 supplement; in DMEM-F12 supplemented with 2 mM L-glutamine, 10% fetal bovine serum and 1% penicillin/streptomycin; or in DMEM-F12 supplemented with 2 mM L-glutamine, 0.5% fetal bovine serum, B27, 50 μM T3, 5 μg/mL Insulin, 5 μg/mL transferrin, 5 ng/mL sodium selenite and 1% penicillin/streptomycin. All specific media used were partially renewed every 3 days, for 10 days (240h) and cells were kept under optimal culture conditions (37 °C and 5% CO_2_). All experiments were carried out at the following time points: 2, 24, 48, 72, 96, 120, 168, and 240 h. All reagents, unless otherwise mentioned, were purchased from Invitrogen-Thermo Fisher Scientific (Carlsbad, CA, USA).

### 2.3. Confocal Fluorescence Microscopy

Confocal fluorescence microscopy was performed for all the cell types and time points used in this study. Briefly, cells were fixed in PBS-paraformaldehyde 4% for 15 min, permeabilized with PBS-triton X100 0.2% for 5 min, blocked with PBS-BSA 5% (Sigma-Aldrich, St. Louis, MO, USA) for 1 h and then incubated overnight at 4 °C with primary antibodies: for neurospheres or dissociated NPC cultures, polyclonal antibody against brain lipid binding protein (BLBP) (Millipore, Merck KGaA, Germany), mouse antibody against nestin (Millipore, Merck KGaA, Germany), polyclonal antibody against the transcription factor SOX2 (SOX2) (Invitrogen, Thermo Fisher, Carlsbad, CA, USA); for cells placed in Neurobasal media, monoclonal antibody against β-tubulin III (Promega Corporation, Madison, WI, USA) was employed; for astrocytes, polyclonal antibody against glial fibrillary acidic protein (GFAP) (Dako, Denmark) was employed; and for oligodendrocytes, monoclonal antibody against oligodendrocyte marker O4 (O4) (R&D Systems, Minneapolis, MN, USA) and polyclonal antibody against myelin basic protein (MBP) (Abcam, UK) were employed. Then, secondary monoclonal and/or polyclonal Alexa Fluor^®^ antibodies conjugated with 546, 568, or 633 nm fluorophores (Molecular Probes Inc, Eugene, OR, USA) were incubated for 2 h together with phalloidin-FITC (commonly used as a cytochemical marker of polymerized actin (F-actin), Molecular Probes Inc, Eugene, OR, USA). Coverslips were mounted on slides and visualized with a HC PL APO 63×/1.40 Oil CS objective lens attached to a Leica TCS-SP5 II confocal microscope (Leica Microsystems, Germany). Images were acquired using the LAS AF 2.2.0 Software (Leica Microsystems, Germany). Quantification analysis of F-actin and GFAP cytoskeleton networks was performed using FibrilTool [24], an ImageJ (National Institutes of Health, USA) plug-in capable of determining the average orientation of a fiber array, providing quantitative information about its anisotropy. The calculations are based on the concept of nematic tensors, used to describe liquid crystals and the mathematical details can be found in [24]. The anisotropy value ranges from a maximum of 1, when all fibers point to the same direction in the array, to a minimum of 0, when they are all randomly oriented. Fluorescence quantifications for GFAP, MBP and O4 were performed using ImageJ 1.8. Briefly, an outline was drawn around each cell and the values for area, the mean grey (fluorescence) value and integrated density (ID) were obtained along with the corresponding measurements for the adjacent background. Then, the corrected total cell fluorescence (CTCF) was calculated for each experimental situation using the following equation:

(1) $$CTCF=ID-\left(A_{c}\times F_{B}\right)$$

where $A_{c}$ is the selected cell area and $F_{B}$ is the mean grey (fluorescence) value of the background.

For GFAP, direct CTCF values were plotted for all experimental conditions. For O4 and MBP, the ratios between O4 CTCF and MBP CTCF were plotted for all experimental conditions.

### 2.4. Optical Tweezers Setup and Calibration

The optical tweezers (OT) system employed an infrared Ytterbium linearly polarized and collimated laser beam with a wavelength of 1064 nm and maximum power of 5 W (model YLR-5-1064-LP) (IPG Photonics, NY, USA). The laser was coupled to an inverted Nikon Eclipse TE300 microscope (Nikon, Melville, NY, USA) equipped with a PLAN APO 100X 1.4 NA DIC H Nikon objective, used to create the trap. The OT system was calibrated using the same procedure previously described [13,25,26]. The trap stiffness (*k_OT_*) per unit power (*P*) at the objective entrance was $\left(0.25\pm 0.01\right)\;\mathrm{pN}\mu m^{-1}{\mathrm{mW}}^{-1}.$

### 2.5. Tether Extraction Experiments with Optical Tweezers

Tether extraction experiments using optical tweezers were performed following the same procedures previously described [7,10,11,12,13]. Briefly, neurospheres and/or dissociated NPCs were plated and allowed to attach to glass bottom dishes, previously coated with 0.01% poly-L-lysine. Neurospheres were allowed to attach only for 2 h prior to experiments. Dissociated NPCs were allowed to attach and differentiate for all time points and conditions, as described above. Then, uncoated polystyrene beads (radius = 1.52 ± 0.02 µm) (Polysciences, Warrington, PA, USA) were added and each one of the glass bottom dishes containing the cells was placed in the OT microscope. A bead was trapped and pressed against a chosen cell for ~5 s, allowing its attachment to the cell surface. The microscope motorized stage (Prior Scientific, Rockland, MA, USA) was then set to move with a controlled velocity (1 µm/s). The sample movement (stage displacement *d*) induced a change in equilibrium position ρ of the trapped bead over time. Movies were collected at a frame rate of 10 frames/second using a Hamamatsu C2400 CCD camera (Hamamatsu, Japan) coupled to a SCION FG7 frame grabber (Scion Corporation, Torrance, CA, USA). Using the trap calibration and the measured bead position displacement (Δρ), acquired by analysis of images extracted from the movie, we obtain the force on the bead:

(2) $$F=k_{OT}\;\Delta \rho \;$$

where the trap stiffness value can be increased or decreased by adjusting the laser beam power. 

**Figure 1:**
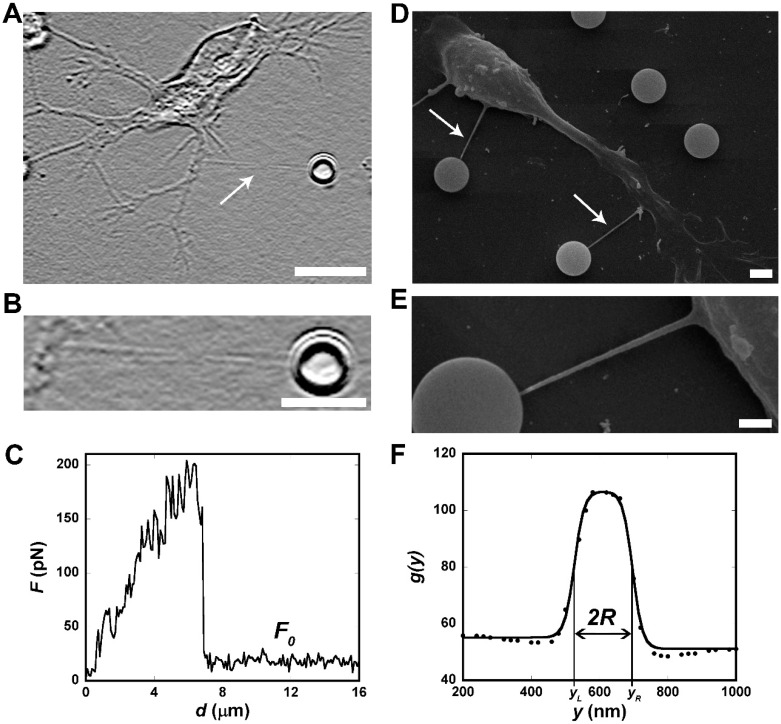
Typical tether force and radius measurements. (**A**) Representative bright field image of a membrane tether extracted from a cell cultured for 48h in Neurobasal medium (indicated by a white arrow). ImageJ shadow north processing filter was applied to better visualize the tether. (**B**) Zoom of the tether in A. Scale bar for A is 10 µm and for B is 5 µm. (**C**) A representative curve of force F versus stage displacement d for a tether extraction experiment. $F_{0}$ is the steady-state tether force. (**D**) SEM representative image of tethers extracted from a cell cultured for 48h in Neurobasal medium. (**E**) Zoom of one of the tethers indicated by the white arrows in D. Scale bar for D is 2 µm and for E is 1 µm. (**F**) Grey level plot profile of the tether in E.

**Figure 2:**
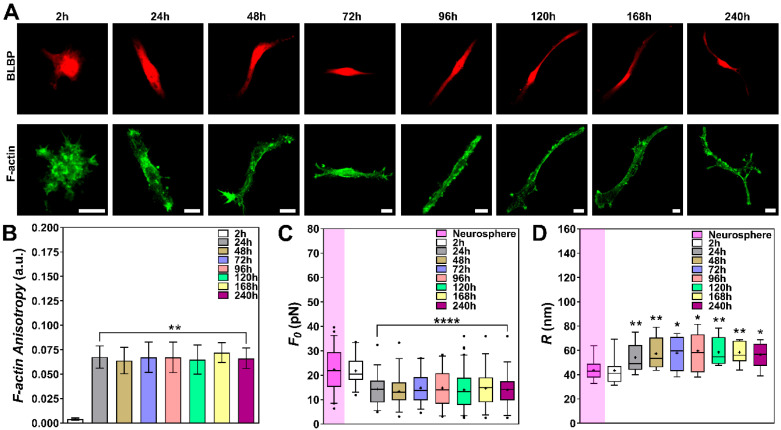
Tether extraction experiments and radius measurements for NPCs. (**A**) Representative images of NPCs stained for BLBP (red) and F-actin, with phalloidin-FITC, (green) from 2 to 240 h. Scale bars are all 10 μm. (**B**) Plot of the mean anisotropy values of F-actin after FibrilTool analysis. At least 10 different cells, for each time point, were imaged and analyzed. They all show similar behaviors as the ones represented in A. Standard errors were used as error bars. (**C**,**D**) Plot of tether force values (at least 32 different cells) (**C**) and tether radius values (at least 12 different cells) (**D**) for each experimental group. The colored boxes extend from 25th to 75th percentiles, with a black horizontal line at the median and a black cross at the mean; black whiskers extend from 5th to 95th percentiles; values outside these ranges are plotted as individual points. Tether force and radius values for cells in neurospheres are also represented (highlighted with a purple rectangle) for comparison purposes. * means *p* < 0.05, ** means *p* < 0.01, **** means *p* < 0.0001 in *Mann-Whitney* *U*-test statistics when comparing each time point with the 2 h condition.

**Figure 3:**
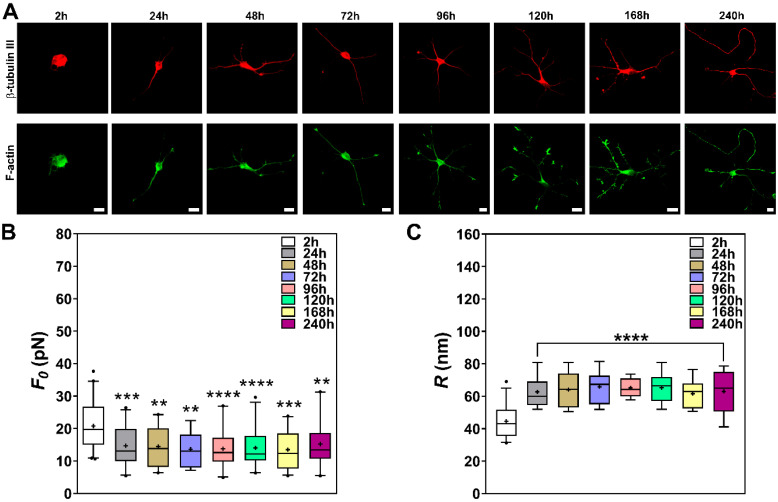
Tether extraction experiments and radius measurements for NPCs cultured in Neurobasal medium. (**A**) Representative images of cells stained for β-tubulin III (red) and F-actin (green) from 2 to 240 h. Scale bars are all 10 μm. (**B**,**C**) Plot of tether force values (at least 18 different cells) (**B**) and tether radius values (at least 10 different cells) (**C**) for each experimental time point. The colored boxes extend from 25th to 75th percentiles, with a black horizontal line at the median and a black cross at the mean; black whiskers extend from 5th to 95th percentiles; values outside these ranges are plotted as individual points. * means *p* < 0.05, ** means *p* < 0.01, *** means *p* < 0.001, **** means *p* < 0.0001 in Mann-Whitney U-test statistics when comparing each time point with the 2 h condition.

**Figure 4:**
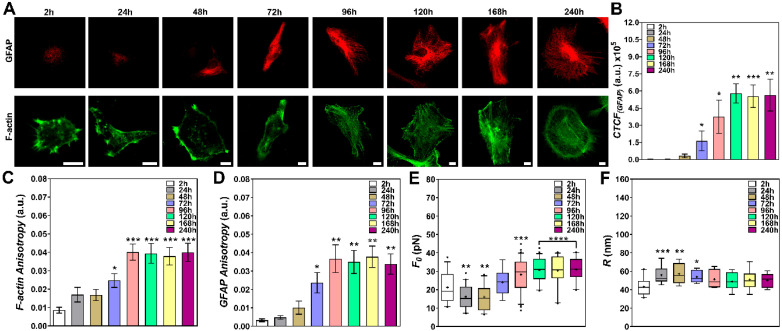
Tether extraction experiments and radius measurements for NPCs induced to differentiate into astrocytes. (**A**) Representative images of cells stained for GFAP (red) and F-actin (green) from 2 to 240 h. Scale bars are all 10 μm. (**B**) Plot of the mean CTCF values for GFAP within 2 h to 240 h of culture. (**C**,**D**) Plot of the mean anisotropy values of F-actin staining (**C**) and GFAP staining (**D**) after FibrilTool analysis. At least 10 different cells for each experimental condition were analyzed. Standard errors were used as error bars in B, C and D. They all show similar behaviors as the ones represented in A. (**E**,**F**) Plots of tether force (at least 16 different cells) (**E**) and tether radius values (at least 10 different cells) (**F**) for each time point. The colored boxes extend from 25th to 75th percentiles, with a black horizontal line at the median and a black cross at the mean; black whiskers extend from 5th to 95th percentiles; values outside these ranges are plotted as individual points. * means *p* < 0.05, ** means *p* < 0.01, *** means *p* < 0.001, **** means *p* < 0.0001 in *Mann-Whitney U*-test statistics when comparing each time point with the 2 h condition.

**Figure 5:**
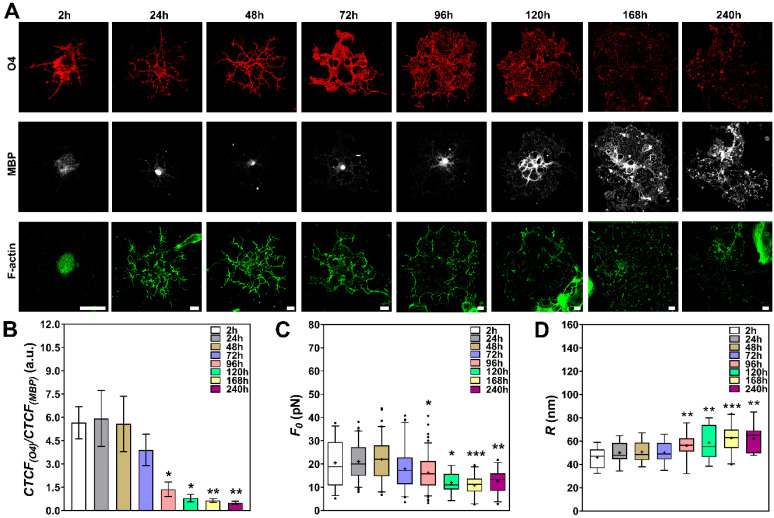
Tether extraction experiments and radius measurements for NPCs induced to differentiate into oligodendrocytes. (**A**) Representative images of cells stained for O4 (red), F-actin (green) and MBP (white) from 2 to 240 h. Scale bars are all 10 μm. (**B**) Ratio of O4 and MBP CTCF values over the course of differentiation from 2 to 240h. At least 10 different cells for each condition were imaged and analyzed in B. They all show similar behaviors as the ones represented in A. (**C**,**D**) Plots of tether force (at least 20 different cells) (**C**) and tether radius values (at least 12 different cells) (**D**) for each condition. The colored boxes extend from 25th to 75th percentiles, with a black horizontal line at the median and a black cross at the mean; black whiskers extend from 5th to 95th percentiles; values outside these ranges are plotted as individual points. * means *p* < 0.05, ** means *p* < 0.01, *** means *p* < 0.001 in *Mann-Whitney U*-test statistics when comparing each time point with the 2h condition.

A representative curve of force F versus stage displacement d for a tether pulling experiment in shown in Figure 1C. The tether force $F_{0}$ referred to as the average value for the steady-state force in Figure 1C, was measured. Then, values of tether force, each obtained from an individual cell, were grouped into different data sets per experimental condition. The tether force plots (Box-and-Whiskers plots) of Figure 2C, Figure 3B, Figure 4E, and Figure 5C represent the distribution of these individual values. Finally, the mean values for tether force $F_{0}$ corresponding to each experimental situation were also obtained. All experiments in the OT microscope were conducted in optimal culture conditions (37 °C and 5% CO_2_). Data analysis and force calculations were performed using ImageJ 1.8 and Kaleidagraph 4.5 (Synergy Software, Essex Junction, VT, USA) software packages.

### 2.6. Measurements of Tether Radii

After extracting the cell membrane tethers, the beads used during the extraction were attached to the coverslip. The samples were then fixed and prepared for SEM following the same procedures previously described [7,10,11,12,13]. Thus, the mean values for tether radii, R, corresponding to each experimental situation were obtained. 

However, the aforementioned procedure could not be employed to measure the tether radii extracted from vesicles that appeared at oligodendrocyte cell surfaces. To circumvent this issue we adopted another previously validated method, already tested for the same purpose [7,13]. This method is based on the force barrier theory for tether formation [27] and its theory gives the following relation:

(3) $$\frac{F_{m\left(ves\right)}}{F_{0\left(ves\right)}}=1+\frac{1}{2}\frac{R_{p\left(ves\right)}}{R_{\left(ves\right)}}\;$$

where $F_{0\left(ves\right)}\;$ is the vesicle tether force, $F_{m\left(ves\right)}$ is the vesicle maximum force before tether formation, $R_{\left(ves\right)}\;$ is the vesicle tether radius and $R_{p\left(ves\right)}$ is the radius of the circular patch contact area between the bead and the vesicle membrane.

**Figure 6:**
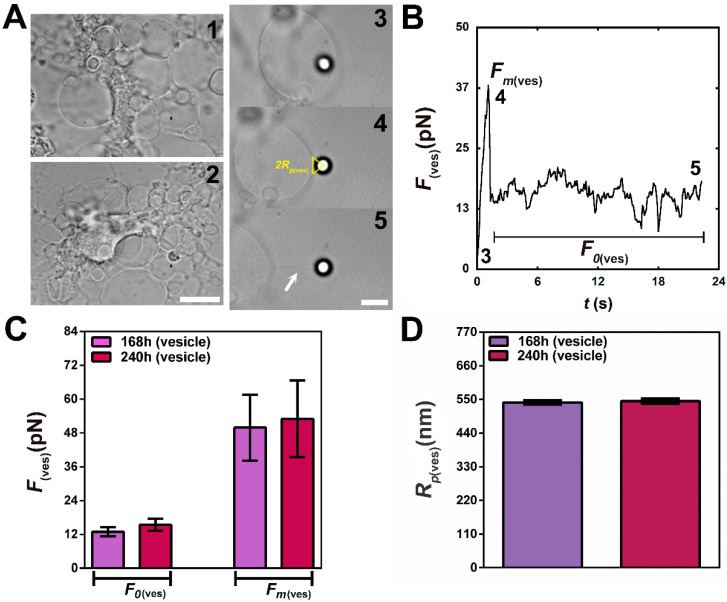
Tether extraction experiments and patch radius measurements for vesicles stemming from oligodendrocyte cell surfaces. (**A**) Typical bright field images of oligodendrocytes at 168 h (Image 1) and 240 h (Image 2), showing several vesicles along their surfaces. (Images 3–5) Selection of images of a typical tether extraction experiment from the surface of a vesicle. (Image 3) Initial moment, bead is being pressed against the vesicle with the optical trap, (Image 4) moment when the force reaches the maximum value – schematics indicating how $R_{p\left(ves\right)}$ is obtained – and, (Image 5) membrane tether already formed, indicated as a white arrow. Scale bar for Images 1 and 2 is 10 μm and for Images 3, 4 and 5 is 5 μm. (**B**) Force curve of a tether extracted from a vesicle. Numbers 3, 4, and 5 in the plot correspond to the images of the same numbers in A. $F_{m\left(ves\right)}$ means the maximum force before tether formation and $F_{0\left(ves\right)}$ means the steady-state tether force. (**C**,**D**) Plot of the mean values of $F_{0\left(ves\right)}$ and (**C**) and $R_{p\left(ves\right)}$ (**D**) for each experimental condition, as indicated by the plot legends. Standard errors were used as error bars in C and D. At least 20 different vesicles were analyzed for each situation.

The values of $F_{m\left(ves\right)}\;$ and $F_{0\left(ves\right)}\;$ were obtained from the force curves, such as the one represented in Figure 6B; $R_{p\left(ves\right)}$ was measured from the image of the bead and the deformed vesicle surface. The value of $R_{\left(ves\right)}$ was then obtained from Equation (3).

### 2.7. Determination of Membrane Elastic Properties

Once the values of $F_{0}\;$ and R were measured, it was then possible to determine the values of the cell membrane tension (CMT, $\sigma_{eff}$) and the cell membrane bending modulus (CMBM, $\kappa_{eff}$). These two parameters are derived from the minimization of the Helfrich–Canham free energy for a cylindrical membrane tether [28,29]. Thus, the CMT ($\sigma_{eff}$) is given by:

(4) $$\sigma_{eff}=\frac{F_{0}}{4\pi R}\;$$

and the CMBM ($\kappa_{eff}$) by:

(5) $$\kappa_{eff}=\frac{F_{0}R}{2\pi }$$

The values of membrane tension, $MT_{\left(ves\right)}$, and bending modulus, $BM_{\left(ves\right)}$, for oligodendrocyte vesicles were also determined using the equations above.

### 2.8. Statistical Analysis

Some data are presented as mean ± standard error. For tether force and radius values, Box-and-Whiskers plots were used. The boxes extend from the 25th to 75th percentiles, with a black horizontal line at the median and a black cross at the mean; black whiskers extend from 5th to 95th percentiles for all experimental conditions; values outside these ranges are plotted as individual points. Data were analyzed using GraphPad Prism statistics software 8.1 (GraphPad Software, Inc. La Jolla, CA, USA). Mann–Whitney *U*-tests were used for comparisons between each situation and the 2 h condition. * means *p* < 0.05; ** means *p* < 0.01; *** means *p* < 0.001 and **** means *p* < 0.0001. The *p*-values and other numbers for all experiments are provided in the figure legends.

## 3. Results

### 3.1. OT as a Tool for Measuring CM Elastic Properties of NPCs and Differentiated Cells

Aiming to characterize the CM elastic properties of NPCs over the course of their distinct differentiation fates, and to compare the results with those found for cells maintained with stem capacity, either as neurospheres or as isolated NPCs, OT-based tether extraction experiments were performed for each cell type over periods of 2–240 h in culture. As an example of the experimental procedures, images associated with a tether extraction experiment performed in a representative cell are shown in Figure 1A (extracted tether is indicated by a white arrow) and Figure 1B (zoom of the extracted tether). The corresponding tether extraction force curve is shown in Figure 1C. The tether force, $F_{0}$, (referred to as the steady-state force in Figure 1C) and the tether radius, R, (Figure 1D–F) were carefully measured for each experimental time point. The results are presented in the following sections.

### 3.2. CM Elastic Properties Measured for NPCs Vary Only in the Initial Hours after Plating

NPCs, previously grown as neurospheres for 5 days (Figure S1A), were dissociated and replated as isolated cells in a culture medium that maintains their stemness. Their morphologies were followed from 2–240 h after plating. Figure 2A displays the F-actin cytoskeleton of these cells (stained in green for phalloidin-FITC) together with BLBP (stained in red), known to be expressed in NPCs during development [30]. Moreover, NPCs were also stained, after 240 h in culture, for nestin (in white) and SOX2 (in red) together with phalloidin-FITC (in green) and the cell nucleus, stained with DAPI (in blue) (Supplementary Figure S1B).

The actin cytoskeleton architecture, with the exception of the 2-h condition, appeared to not present any substantial variations throughout the days of culture, as verified by the images (Figure 2A). However, to better quantify these visual observations, we also performed quantitative analysis using FibrilTool [24]. The results of this analysis (Figure 2B) showed that, overall, the cells present a similar F-actin anisotropy throughout the entire experiment, except for the cells at the 2-h time point.

Next, tether extractions were performed, for each time point, on dissociated NPCs maintained in culture and with morphologies similar to those of Figure 2A. The corresponding values of tether force are shown in Figure 2C. No statistically significant change in tether force values were found for NPCs from 24 to 240 h in culture (Figure 2C). However, a ~1.5-fold decrease in tether force value (from 22 ± 1 to estimated 14 ± 1 pN) was found when comparing the 2-h condition with all the other ones (Figure 2C).

The tether radii were also measured using SEM, for each time point, and from cells with morphologies similar to those of Figure 2A. Figure 2D shows the values for the NPCs from 2 to 240 h in culture. No statistically significant change in tether radius values were found for NPCs from 24 to 240 h (Figure 2D). However, a ~1.3-fold increase in tether radius value (from 43 ± 3 to estimated 57 ± 4 nm) was found when comparing the 2-h condition with all the other ones (Figure 2D).

In order to compare the CM elastic properties of dissociated NPCs versus the ones in spheres, we cultured neurospheres for five days (Supplementary Figure S1A), replated and allowed them to attach for 2h in culture medium to maintain their stemness (Supplementary Figure S1A). Tether force and radius measurements were also performed on cells around the neurospheres. Their mean values of $F_{0}\;$ and R  are also shown in Figure 2C,D, respectively. No statistically significant changes in tether force and radius values were found between the 2-h-dissociated NPCs and cells from the neurospheres. However, the similar ~1.5-fold decrease in tether force value and the ~1.3-fold increase in tether radius were still evident when comparing the neurosphere condition with the 24–240-h conditions.

**Figure 7:**
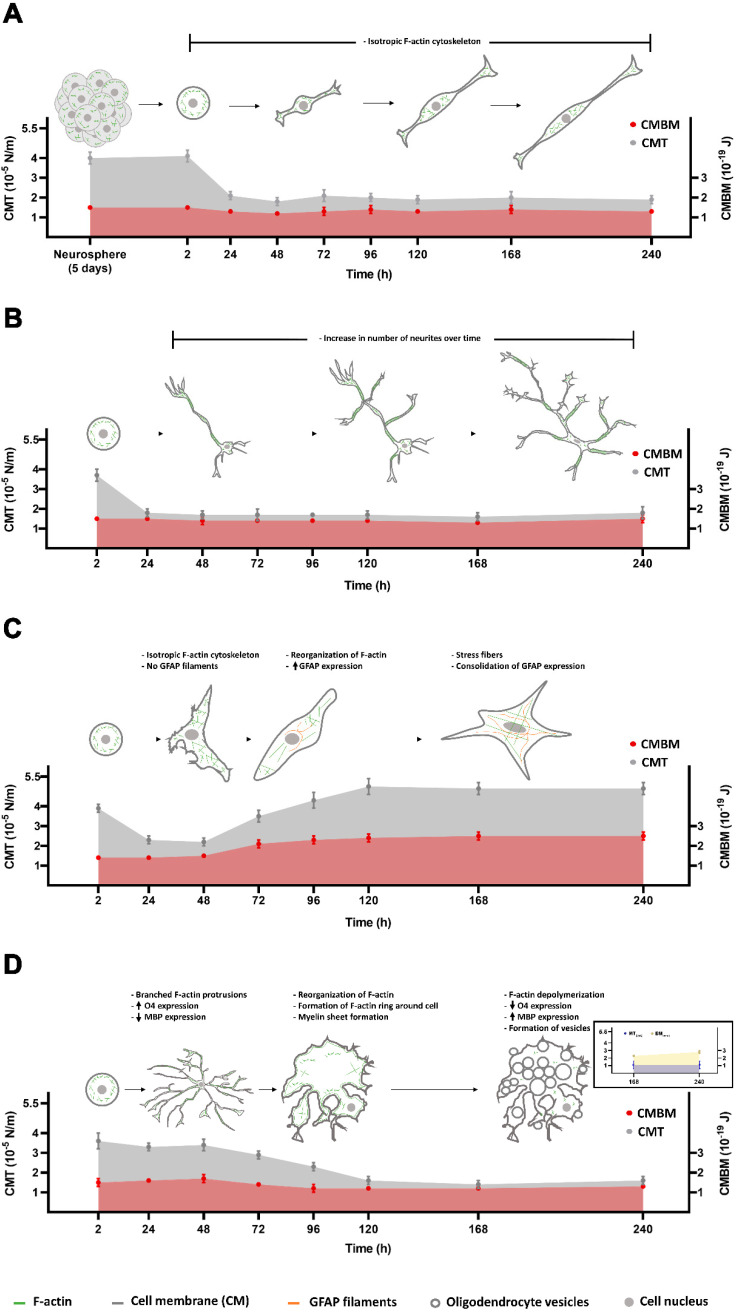
Schematic representation correlating the variations in morphological phenotype with CMT and CMBM for (**A**) undifferentiated NPCs, (**B**) NPCs plated in Neurobasal medium, (**C**) NPCs induced to differentiate into astrocytes and (**D**) NPCs induced to differentiate into oligodendrocytes. Insert in D represents the membrane tension and bending modulus for vesicles that appear at the surface of oligodendrocyte cells.

Taken together, the results confirm that NPCs maintain their stemness over the entire experiment, with an initial two-fold drop in their CMT values in the very early hours (from 2 to 24 h), followed by stabilization in the subsequent hours (from 24 to 240 h) (Figure 7A, grey curve and dots). The CMBM values, on the other hand, remain constant over time (Figure 7A, red curve and dots). These variations in CMT are not attributed to the dissociation of neurospheres but may be related to cell spreading and the acquisition of another morphological phenotype.

### 3.3. CM Elastic Properties Measured for Cells Cultured in Neurobasal Medium Show a Similar Pattern in Comparison to NPCs

When dissociated, some NPCs were plated in a culture dish containing Neurobasal medium, commonly known as a medium used to maintain neurons in culture. Again, the cell morphology was followed from 2 to 240 h after plating. Figure 3A shows the phalloidin-FITC labeled F-actin cytoskeleton (in green), together with β-tubulin III (in red). The results show that the cells, after 24 h, acquired a neuronal-like morphological phenotype, displaying neurites and growth cones that increased in numbers within the subsequent hours of culture (Figure 3A).

OT-based tether extractions, for each time point, were always performed in cells with morphologies similar to those of Figure 3A. The mean values of $F_{0}\;$ are shown in Figure 3B. No changes in tether force values were found for cells from 24 to 240 h in culture (Figure 3B). However, a ~1.5-fold decrease in tether force (from 21 ± 1 to estimated 14 ± 1 pN) was found when comparing the 2-h condition with all other ones (Figure 3B).

Regarding the tether radii, no statistically significant change was detected among the time points ranging from 24 to 240 h in culture (Figure 3C). However, a ~1.4-fold increase in tether radius (from 45 ± 2 to estimated 64 ± 3 nm) was found when comparing the 2-h condition with all the other ones (Figure 3C).

In view of the prior demonstration that, regardless of the neuronal region (cell body, neurites and growth cones) there is no significant difference in the tether force and radius [13], we chose to pool all the measurement results together in the plots (Figure 3B,C). The values found for tether force and radius were also of the same order of magnitude as those previously found for mouse cortical and ganglionic eminence neurons [13].

The results confirm that NPCs are able to acquire, after 24h, a morphological phenotype associated with neurons, showing an initial 2.1-fold drop in their CMT values in the very early hours, followed by stabilization in subsequent hours (from 24 to 240 h), while increasing the number and length of neurites and number of growth cones over time. The CMBM values, on the other hand, do not vary during the experimental time points. Finally, the values for the CMT (Figure 7B, grey curve and dots) and CMBM (Figure 7B, red curve and dots) are very similar to those found for NPCs (Figure 7A).

### 3.4. The Differentiation Process for Astrocytes Reveals Interesting Patterns that Correlate Cytoskeletal Architecture Remodeling with Changes in CM Elastic Properties

Dissociated NPCs were also induced to differentiate into astrocytes. The cell morphology was followed from 2 to 240 h after plating. Figure 4A shows the actin cytoskeleton, stained for phalloidin-FITC (in green), together with GFAP (in red) [31]. The results demonstrate that even though the cells acquired a putative astrocyte morphology within the first hours, the expression of GFAP began showing up as filaments, in some cells, only 48 h after plating, yielding more stable filaments after 72 h of culture (Figure 4A,B). Interestingly, the actin cytoskeleton also presented a morphological shift, from small actin filaments (in the first 48–72 h) to very well-defined stress fibers after 96 h (Figure 4A). These results were confirmed by the FibrilTool analysis, which showed that the anisotropy of both actin and GFAP cytoskeletal networks increased with time in culture (Figure 4C,D).

Tether extraction measurements, for each time point, were always performed in cells with morphologies similar to those indicated in Figure 4A. The values of tether force are shown in Figure 4E. A clear decrease (~1.3-fold) in tether force was observed from 2 to 48 h, followed by a slight recovery at 72 h after plating, consistently correlated with the increase in GFAP expression and the shift in F-actin architecture described above (Figure 4A–D). Moreover, the tether force values found after 96 h (~30 pN) increased ~1.4-fold when compared with the 2-h result and also increased almost two-fold when compared with the 24–4-h conditions (Figure 4E).

Figure 4F shows the tether radius values. A clear increase (~1.3-fold) in values from 24 to 72 h and a slight increase from 96 to 240 h (~1.16-fold) when compared to the 2-h time point were also observed (Figure 4F).

Altogether, the results confirm that NPCs differentiate into astrocytes after 48–72 h. The shift in actin cytoskeleton architecture together with the increase in GFAP expression are both correlated with the changes in CM elastic properties observed for these cells. The results show that there is a ~1.3-fold increase in CMT (Figure 7C, grey curve and dots) and a ~1.7-fold increase in CMBM (Figure 7C, red curve and dots) in differentiated astrocytes (96–240h) when compared to NPCs (Figure 7A).

The values of CMT and CMBM found for astrocytes at advanced culture stages are within the same order of magnitude of those previously found for differentiated astrocytes obtained from newborn mice [13].

### 3.5. The Differentiation Process for Oligodendrocytes Reveals Interesting Patterns that Correlate Cytoskeletal Remodeling and Expression of Specific Markers with Changes in CM Elastic Properties

Finally, NPCs were also induced to differentiate into oligodendrocytes. Likewise, we followed the cell morphology during the process. Figure 5A shows the actin cytoskeleton (in green), together with two specific markers for oligodendrocytes: O4 (in red) and MBP (in white) [32]. Interestingly, the expression of O4 increased in the first hours of culture and then decreased after the 96-h time point. Conversely, the expression of MBP, which was low in the first hours, increased in the last hours, especially after the 96-h time point (Figure 5A). The antagonistic expression patterns between O4 and MBP become more evident in Figure 5B, where the ratios between O4 and MBP fluorescence intensities were plotted. The cell morphology and actin cytoskeleton organization also dynamically changed over time in culture. Cells initially presented a star-like branched morphology during the first 48 h, with several F-actin containing protrusions (Figure 5A). However, this ramified morphology changed to a more flat-like lamellar shape, with the formation of membrane extensions, after 72–96 h. These changes were correlated with the increase in MBP expression and the simultaneous decrease in O4 expression. Interestingly, in the same time frame, the actin cytoskeleton also presented peculiar changes in its architecture. These changes first appeared in the outermost region of the membrane extensions surrounding the cell as a sort of ring and then disappeared in the subsequent hours (168 h and 240 h) (Figure 5A). This morphological behavior had already been documented in other studies [33,34,35]. 

Tether extraction experiments, for each time point, were performed in cells with similar morphologies as those represented in Figure 5A. The values of $F_{0}$ are shown in Figure 5C. No differences in tether force were observed in the first 72 h as compared to the 2-h time point (Figure 5C). However, a statistically significant decrease in tether force was observed in time points between 96 h and 240 h (Figure 5C). This result can be well correlated with the increase in MBP expression, the change in cell morphology and the shift in actin cytoskeleton architecture described above (Figure 5A). The values for 120, 168, and 240 h (on the order of ~12 pN of force) were ~1.7 times lower than those found within the first 48 h (on the order of ~21 pN of force).

Concerning the tether radius values, Figure 5D depicts how these numbers changed along the oligodendrocyte differentiation process. We observed a clear increase (~1.3-fold) in tether radii from 120 to 240 h when compared to the 2-h condition (Figure 5D).

Another feature that appeared in the last hours of culture (168–240 h) was the presence of spherical vesicle-like membrane protrusions stemming from the surface of oligodendrocytes (Figure 6A, images 1 and 2). These vesicles were previously documented several years ago in ultrastructural characterizations of cultured oligodendrocytes [36], but have apparently been neglected since that time. 

In order to explore the elastic properties of these vesicles and to compare the results with those obtained directly from the cell surface, we next extracted tethers from these vesicles (Figure 6). Examples of these vesicles are shown in Figure 6A, with bright field images of three different situations: (image 3) when the bead is attached to the vesicle surface, (image 4) when the force is the maximum force $F_{m\left(ves\right)}$ (before tether formation), and (image 5) when the tether is already formed and the measured force is the steady-state tether force $F_{0\left(ves\right)}$. Figure 6B shows the force curve, with the numbered points corresponding to the numbered images in Figure 6A (images 3, 4 and 5). Figure 6C represents the mean values for $F_{m\left(ves\right)}$ and $F_{0\left(ves\right)}$ at 168 and 240 h of culture. No differences among force values were observed for both time conditions. The values found were 14 ± 2 pN and 15 ± 2 pN for $F_{0\left(ves\right)}\;$ and 50 ± 12 pN and 53 ± 13 pN for $F_{m\left(ves\right)}$, respectively at 168- and 240-h conditions. The bead/membrane contact patch radius, $R_{p\left(ves\right)}$, was also measured by image analysis. Figure 6A (image 4) represents an example of a chosen frame and indicates how $R_{p\left(ves\right)}$ was obtained in that case. $R_{p\left(ves\right)}$ was measured for both conditions (168 and 240 h). No statistically significant differences were observed (Figure 6D). The values found were 540 ± 7 nm and 545 ± 8 nm, respectively for the 168 and 240 h conditions. Using the values of $R_{p\left(ves\right)}$, $F_{m\left(ves\right)}$, and $F_{0\left(ves\right)}$, we calculated the mean tether radius values of these vesicles, $R_{\left(ves\right)}$, using equation (3). The values found were 104 ± 36 nm and 112 ± 44 nm, respectively for the 168- and 240-h conditions. Finally, the membrane tension, $MT_{\left(ves\right)}$, and bending modulus, $BM_{\left(ves\right)}$, for vesicles were calculated.

Altogether, the results confirm that NPCs are able to differentiate into oligodendrocytes. Although O4 appeared in the first hours of induction, MBP expression only appeared in very late hours. The change in actin cytoskeleton architecture, the increase in MBP expression, and the formation of vesicles around the surface of oligodendrocytes are all correlated with the decrease in CMT observed for these cells after 120 h and until 240 h (Figure 7D, grey curve and dots). The CMBM values (Figure 7D, red curve and dots) do not vary significantly. Finally, vesicles that appeared at the surface of oligodendrocytes at 168 and 240 h present similar membrane tension values but higher bending modulus values when compared to those found for the cell surface (Figure 7D insert, yellow and blue curves and dots).

## 4. Discussion

The elastic properties of cell surfaces are increasingly recognized as key regulators of cell functions [1,14,16,37,38]. Here, we have mapped the variations in NPC membrane elastic properties along their distinct differentiation fates. The results show that these variations are correlated with striking morphological phenotype changes that occur with NPCs. We conjecture that these alterations could affect the differentiation process and ultimately the cell functions.

CMT has been considered an ideal candidate for global cell signaling, in view of its fast equalization around the cell in response to a stimulus [14], although controversies exist [39]. Conversely, CMBM is locally adjustable by a variety of mechanisms and is important for dynamic cell remodeling and movement [4]. These considerations emphasize the need to precisely determine CMT and CMBM for different cell types and in different biological contexts. Tether pulling experiments are a reliable choice method for such purpose [14]. However, it is important to measure not only the tether force but also the tether radius. Tether force can be measured with OT [6,7] or AFM [8,9]. However, the measurement of tether radii is a challenge, as they are typically below the resolving limit of conventional optical microscopes. Some studies just assume a standard value for the CMBM (~0.27 pN.µm—first obtained by [40]) and calculate CMT from direct measurements of tether force alone. This assumption that CMBM has a quasi-universal value for all cell membranes is unwarranted as CMBM itself is also dependent on tether force and radius [28,29]. Therefore, a previously established correlative microscopy-based method [7,13] was used in the present study. In this method, one extracts a tether and measures its force with OT and its radius with SEM. The intrinsic difficulty in performing the method is the main obstacle for this technique. Novel super-resolution live-microscopy techniques, capable of measuring simultaneously the tether radius and force, should be undertaken to move the field forward. Non-invasive methods have been recently developed [41,42] but further tests remain necessary before they may be used as standards. Therefore, even if there might be some difficulties related to the correlative experiments performed in this work, they are still the gold-standard method to precisely determine the elastic properties of cell membranes.

Previous studies have performed tether extraction measurements from different precursor cell types, such as human mesenchymal stem cells [43,44], mouse embryonic stem cells [45,46], and even mouse neural stem cells [47]. However, to the best of our knowledge, the present study is the first to measure both the steady-state tether force and tether radius for precise determination of the CM elastic properties of murine NPCs over the course of their differentiation. We not only measured and mapped the changes in elastic properties of NPCs during differentiation, but also correlated these changes with morphological phenotype modifications acquired by these cells along the process.

Mouse NPCs used in this study have an embryonic origin and presented a low-density and isotropic actin network over the course of the entire experiment. This feature was previously documented for mouse embryonic stem cells [48] and conjectured to be necessary to maintain the balance between cortical and nuclear stiffness such that the rigidity of the cortex never exceeds the rigidity of the nucleus. This is important because, otherwise, the nucleus would end up sensing the cell’s own stiff cortex instead of the external mechanical environment. It is well established that nuclear stiffening follows stem cell differentiation [49]. The observed low-density isotropic actin network is correlated with low elastic constant values, as observed in the present study, suggesting that mouse NPCs have a weak interaction between the plasma membrane and the adjacent cortical cytoskeleton. Indeed, these cells must be prepared to give rise to the three cell types studied herein. Therefore, NPCs low CM elastic constants and less structured actin cytoskeleton can be important for allowing fast conversion into fully developed differentiated cells. More recently, researchers have shown in mice that neural stem cells can contribute to embryonic, early postnatal and adult neurogenesis in the hippocampus, and that these cells are continuously generated throughout a lifetime [50], which reinforces the importance of determining the CM elastic properties of NPCs. 

In this study, we plated neurosphere-dissociated cells in Neurobasal medium. Cells displaying typical neuronal morphologies (with cell bodies, neurites and growth cones) were observed after 24 h. However, we also identified β-tubulin III labeled cells following the first 2 h after plating and this might be attributed to the embryonic stage employed to obtain the NPCs: the neurogenesis window [17], which attains its peak between 12 and 16 days of embryonic development in mice. In this stage, not only neural stem/progenitor cells but also neuron-restricted progenitors and differentiated neurons are present and this might explain the very early β-tubulin III observed phenotype (Figure 2A). Thus, overall, we do not have quite enough evidence to categorically state that NPCs are differentiated into neurons, although the observed morphological phenotype is quite typical (especially for cells from 24 to 240 h). In contrast, the CM elastic properties of these cells were low and remained constant for almost the entire experiment (from 24 to 240 h). Similar mechanical features have been previously described for differentiated mouse neurons [13]. The fact that these cells present low elastic constant values may also be related to a weak interaction between the plasma membrane and the cortical cytoskeleton.

Moreover, we also observed that CM elastic properties, particularly CMT, changed dramatically in the first hours (from 2 to 24 h) for most experimental conditions. Although the tether force measurements were performed in the overall cell populations, lacking biomarkers for NPCs, we take it as a plausible indicator that the changes in CMT are probably correlated with the cell spreading behavior, that changes from initially round cells (grown in spheres) to a final state in which the cells are fully spread/attached within the first 24 h of experiment. Though never demonstrated before during neural differentiation, this behavior has been previously described for fibroblasts [51]. The CMT decreased during spreading and it was assumed that the addition of new membrane and the increase in membrane area were mainly responsible for such phenomenon [51]. Moreover, using embryonic stem cells, two different groups have described, in quite recent studies yet to be published, that the decrease in CMT occurred when embryonic stem cells changed from their round and naïve state to a spread and primed state, and that the observed decrease in CMT was correlated with a decrease in membrane-cytoskeleton attachment [45,46] via GSK3β-driven β-catenin degradation [46], which in turn controls membrane tension and allows exit from naïve pluripotency. Our results are in agreement with all the above descriptions, even if the murine NPCs used in this study no longer display the transition between naïve and primed states. Moreover, all mentioned studies [45,46,51] assume a standard value for the CMBM and calculate CMT from direct measurements of tether force, without measuring the tether radius. As we have discussed, one should be careful when assuming that CMBM has a quasi-universal value for all cell membranes, since it depends on both tether force and radius [28,29]. In line with this, the results of the present study and previous observations [7,10,13] confirm that CMBM can vary depending on the cell type and cell context.

We also induced NPCs to differentiate into astrocytes. Moreover, we noticed that the in vitro differentiation occurred more slowly, possibly owing to the fact that NPCs were isolated during the neurogenesis peak, between 12 and 16 days of murine embryonic development [17], while gliogenesis occurs only in late-gestation to perinatal periods [21]. Thus, cells with an astrocytic phenotype only appeared around 48–72 h of differentiation after the consolidation of GFAP expression. The increase in CM elastic properties values followed the consolidation of GFAP expression and actin cytoskeleton reorganization that strikingly changed from a low-density isotropic meshwork to a more organized stress fiber pattern. 

Lastly, we also induced NPCs to differentiate into oligodendrocytes and similarly to astrocytes, the in vitro differentiation process also occurred more slowly. This behavior partially recalls what happens during mouse embryonic development. Although oligodendrocyte progenitor cells are generated from NPCs in different locations and times during CNS development, the formation of mature myelinated oligodendrocytes only occurs around 5–7 days after birth in mice [32]. In our in vitro differentiation model, cells with the mature oligodendrocyte phenotype appeared around 120 h after plating. This differentiation coincided with a decrease in O4 and an increase in MBP expression levels, and it correlated with a striking actin cytoskeleton reorganization, changing from initially actin-rich tubular protrusions to a more lamellar morphology, with actin moving towards the cell periphery until almost disappearing at later stages. This morphological behavior has already been documented in other studies [33,34,35]. Moreover, Nawaz et al. [34] performed tether pulling experiments in oligodendrocytes after being in culture for 48 (without myelin) and 120 h (with myelin sheath). They found a ~1.3-fold decrease in tether force, similarly to that found in our measurements. However, again, these authors did not measure the tether radius but concluded, only from the tether force, that the CMT was decreasing. This decrease was associated with actin depolymerization via ADF/cofilin and induced membrane spreading and myelin sheet growth [34]. We performed tether force and radius measurements for each experimental time point ranging from 2 to 240 h of differentiation and demonstrated that CMT was indeed decreasing, while CMBM did not vary. Another feature that appeared in the last hours of culture (168 and 240 h) was the presence of membrane vesicles at the surface of oligodendrocytes. These vesicles do not appear to be apoptotic bodies as their sizes can reach up to 4–5 times those described for apoptotic vesicles [52,53]. In addition, the oligodendrocyte cells at later culture stages (168 and 240 h) do not appear to have the typical apoptotic morphology (i.e., cell shrinkage followed by cell rounding) [52]. Indeed, these membrane vesicles were previously documented several years ago in ultrastructural characterizations of cultured oligodendrocytes [36], but have been neglected since that time. The formation of these vesicles can be well explained based on the excess membrane production and the huge actin depolymerization associated with an increase in membrane-cortex detachment already described at latter stages of oligodendrocyte differentiation [33,34,35]. The elastic properties of these membrane vesicles were determined and compared with the elastic properties obtained directly from the surface of oligodendrocytes at these time points. The vesicle membrane tension was slightly lower when compared to the CMT but the vesicle bending modulus increased ~2.3-fold when compared to the CMBM. These differences may be associated with the total decoupling of the plasma membrane from the underneath cortical cytoskeleton during vesicle formation. It is noteworthy that, even with the increase in actin depolymerization, the plasma membrane of oligodendrocytes still has some connection with the cortical cytoskeleton, but this connection may be lost during vesicle formation.

In conclusion, we have measured the NPC membrane elastic constants over the course of their distinct differentiation fates. All tether radii and forces were measured. The values, determined under uniform and controlled experimental conditions, not only reinforce confidence but can also be taken as data for future studies. The importance of CMT and CMBM is now established for a variety of cells. However, the mechanisms allowing cells to set and regulate their CM elastic properties remain to be elucidated. The present study contributes to the knowledge that the CM elastic constants not only differ among cell types, but also depend on the cell state at the time of measurement. A variety of cellular processes are already known to affect the CM elastic properties, but how they all manage to determine and maintain their values remains a challenging question for the field.

## Supplementary Materials

The following are available online at https://www.mdpi.com/2073-4409/9/6/1323/s1, Figure S1. NPCs are present in our culture systems. (A) Phase contrast image of neurospheres in culture and two representative neurospheres, one stained for BLBP (in magenta) and another stained for nestin (in white) and SOX2 (in red) together with DAPI (insert in blue). Scale bars are respectively 100 µm, 50 µm and 50 µm. (B) Representative image of a dissociated NPC after 240 h in culture stained for nestin (in white) and SOX2 (in red) together with DAPI (in blue) and actin, with phalloidin-FITC (in green). Scale bar is 20 µm.
