# Supplementary figures and images for: Membrane Elastic Properties during Neural Precursor Cell Differentiation
Source: Cells. 2020 May 26;9(6):1323. doi: 10.3390/cells9061323 (PMC7349228; doi:10.3390/cells9061323)

**A****Culture**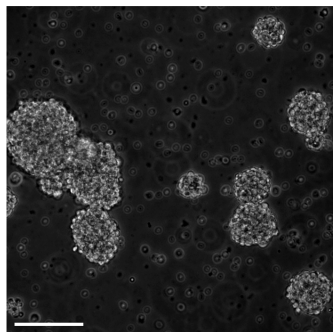**BLBP**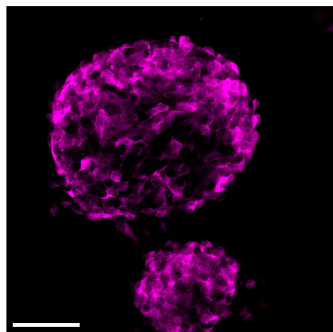**Nestin**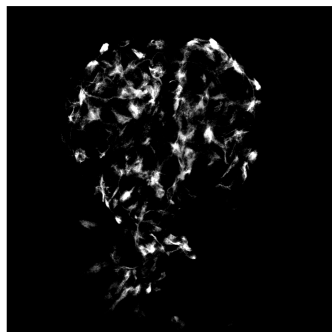**SOX2**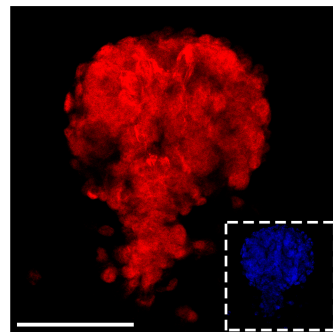**B****DAPI**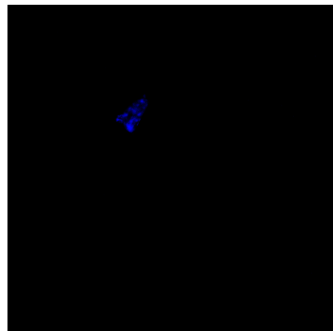**Actin**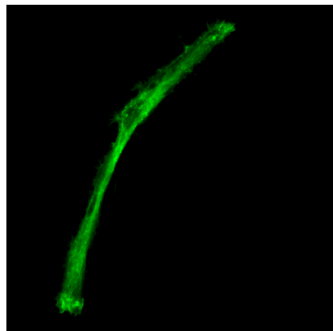**Nestin**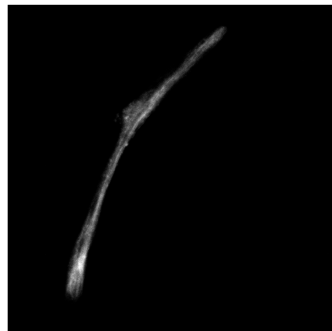**SOX2**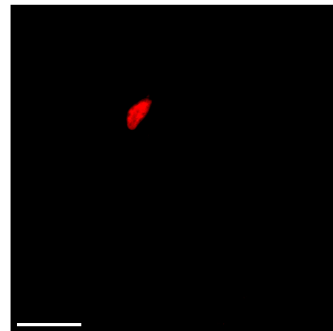

Supplement: Supplementary file 1 [file cells-09-01323-s001.pdf]
